# Automatic Detection of Occluded Main Coronary Arteries of NSTEMI Patients with MI-MS ConvMixer + WSSE Without CAG

**DOI:** 10.3390/diagnostics15030347

**Published:** 2025-02-02

**Authors:** Mehmet Cagri Goktekin, Evrim Gul, Tolga Çakmak, Fatih Demir, Mehmet Ali Kobat, Yaman Akbulut, Ömer Işık, Zehra Kadiroğlu, Kürşat Demir, Abdulkadir Şengür

**Affiliations:** 1 Emergency Medicine Department, School of Medicine, Firat University, Elazig 23119, Turkey; mc.goktekin@firat.edu.tr (M.C.G.); egul@firat.edu.tr (E.G.); 2 Clinics of Cardiology, Balıkesir Atatürk City Hospital, Balıkesir 10050, Turkey; tolgacakmak85@gmail.com; 3 Software Department, Engineering Faculty, Firat University, Elazig 23119, Turkey; 4 Cardiology Department, Medicine Faculty, Firat University, Elazig 23119, Turkey; mkobat@firat.edu.tr (M.A.K.); drr.omr@gmail.com (Ö.I.); 5 Software Department, Technology Faculty, Firat University, Elazig 23119, Turkey; yamanakbulut@firat.edu.tr; 6 Electrical-Electronic Department, Technology Faculty, Firat University, Elazig 23119, Turkey; zehrakad@gmail.com (Z.K.); ksengur@firat.edu.tr (A.Ş.); 7 Artificial Intelligence Department, Firat University, Elazig 23119, Turkey; kursatdemir62@gmail.com

**Keywords:** NSTEMI, main coronary arteries, MI-MS ConvMixer, WSSE, classification

## Abstract

**Background/Objectives:** Heart attacks are the leading cause of death in the world. There are two important classes of heart attack: ST-segment Elevation Myocardial Infarction (STEMI) and Non-ST-segment Elevation Myocardial Infarction (NSTEMI) patient groups. While the STEMI group has a higher mortality rate in the short term, the NSTEMI group is considered more dangerous and insidious in the long term. Blocked coronary arteries can be predicted from ECG signals in STEMI patients but not in NSTEMI patients. Therefore, coronary angiography (CAG) is inevitable for these patients. However, in the elderly and some patients with chronic diseases, if there is a single blockage, the CAG procedure poses a risk, so medication may be preferred. In this study, a novel deep learning-based approach is used to automatically detect the occluded main coronary artery or arteries in NSTEMI patients. For this purpose, a new seven-class dataset was created with expert cardiologists. **Methods**: A new Multi Input-Multi Scale (MI-MS) ConvMixer model was developed for automatic detection. The MI-MS ConvMixer model allows simultaneous training of 12-channel ECG data and highlights different regions of the data at different scales. In addition, the ConMixer structure provides high classification performance without increasing the complexity of the model. Moreover, to maximise the classifier performance, the WSSE algorithm was developed to adjust the classification prediction value according to the feature importance weights. **Results:** This algorithm improves the SVM classifier performance. The features extracted from this model were classified with the WSSE algorithm, and an accuracy of 88.72% was achieved. **Conclusions**: This study demonstrates the potential of the MI-MS ConvMixer model in advancing ECG signal classification for diagnosing coronary artery diseases, offering a promising tool for real-time, automated analysis in clinical settings. The findings highlight the model’s ability to achieve high sensitivity, specificity, and precision, which could significantly improve.

## 1. Introduction

Heart attack is one of the greatest prevalent causes of mortality in the world, and one of the severest sequelae of the cardiovascular disease burden is its appearance in healthy members of the general population in both the developed and developing worlds [1]. Identifying a heart attack and acting on it quickly and correctly can do wonders in terms of survival rate. “Without an early diagnosis, there can also be permanent damage to the heart muscle, impairment of quality of life, and risk of death”. Thus, knowledge of heart attack pathophysiology and ways to detect heart attack results in the design of effective treatment strategies [2].

STEMI (ST-Elevation Myocardial Infarction) is a type of acute myocardial infarction (heart attack) that is associated with a significant elevation of the ST segment on the electrocardiogram (ECG). This generally means that the coronary arteries are fully blocked and immediate action is necessary. Without treatment, a significant portion of the heart muscle can be rapidly injured [3,4].

Non-STEMI (NSTEMI) is a less severe variety of myocardial infarctions, characterized by the absence of a raised ST segment coupled with elevated biomarkers. This symptom is a sign of partial blockage of the coronary arteries and typically results in less extensive damage to the heart muscle. On the other hand, NONSTEMI patients also bear significant cardiovascular risks long-term [5].

By analyzing ECG data, it is possible to identify the occlusions of the main coronary arteries of STEMI patients. Prominent electrocardiograph (ECG) signs for STEMI include ST segment elevations and T wave inversions or Q wave changes. This can assist with locating the completely occluded artery. Anterior STEMI, for example, usually signals left anterior descending artery (LAD) occlusion [6].

In NSTEMI patients, the ECG findings are much more nuanced. ST segment depressions or T wave changes can be present and, alone, are not diagnostic either. So, in the case of NSTEMI, there is high-sensitivity troponin and other clinical data usually adjunct with the ECG. Nevertheless, it is complicated to detect complete main coronary occlusion in NSTEMI patients without coronary angiography because of the tiny nuances in the ECG signal that are too small to be visible [7]. However, the CAG procedure, by its very nature, has significant risks, especially in the elderly and in some chronic diseases. One worry is the risk of kidney injury from the contrast agents used in the procedure. Patients who have conditions like diabetes or high blood pressure are especially at risk for contrast-induced kidney damage, which can worsen kidney function. Moreover, older adults face a greater chance of bleeding or problems with blood vessels at the site where the catheter is inserted, due to fragile arteries and the possible use of blood thinners. The procedure can lead to heart issues, like irregular heartbeats or even heart attacks, particularly in those with serious heart problems [8]. The stress and invasive nature of angiography can worsen existing heart conditions. There is also a risk of infection, especially in older patients who may have weakened immune systems. Recovery can take longer and be tougher for these individuals than for younger patients. Additionally, there can be mental effects. Anxiety or issues with thinking, like confusion, may occur during or after the procedure, especially in patients with past neurological or mental health problems [9,10,11]. Due to these risks of the CAG procedure, in this study, we tried to detect the blocked main coronary arteries of NSTEMI patients without CAG using an innovative deep-learning model using only ECG signals. Thus, if the physicians deem it appropriate, treatment with medication can be applied to the patient group that is at risk of CAG [12].

For the literature studies examined in line with the proposed study, research was carried out with keywords such as ECG, classification and prediction of heart diseases, and occluded coronary artery classification. The studies in the following paragraph are diversified accordingly. There are many existing studies in the same genre, but we have tried to cover the ones that are different in terms of dataset, methodology, and topics covered, and we have included current studies [13,14,15].

When the literature is analysed, some of the existing methods have been carried out by directly using ECG signals in artificial intelligence models. Malakouti [16] chose to use machine learning-based algorithms to distinguish a case of heart disease from ECG signals. First, the characteristic features of ECG signals such as slope and peak are extracted. Using these features, Gaussian Naive Bayes (NB), Random Forest, Logistic Regression, Linear Discriminate Analysis, and Dummy algorithms were used for classification. The Gaussian NB algorithm detected a patient with heart disease from ECG features with a 96% classification accuracy. Baloglu et al. [17] used 12-channel ECG signals to detect the occurrence of myocardial infarction with a customized CNN model. The CNN model is designed to work with 10 layers and one-dimensional data. With this approach, 99.78% classification accuracy was achieved. Khatar and Bentaleb [18] devised a novel method for precisely categorizing cardiac conditions like MI and cardiac arrhythmia (CA). This research employs a novel transformation technique. An RGB image’s R, G, and B channels are mapped to ECG signals’ temporal, frequency-based, statistical, and spatial characteristics. Following this mapping process, the data were put into the ResNet architecture. This model produced classification accuracies of 99.21% for CA and 99.25% for MI. Yousuf et al. [19] used lead II of the ECG to automate the detection of MI. In this study, the PTB dataset in the PhysioNet database was used. Gramian angular sum field (GASF) and Gramian angular difference field (GADF) algorithms are used to obtain robust 2D representations of the ECG signal. After a pre-processing step, the 2D data were fed into a light-weighted CNN model with six layers. This approach achieved an average classification accuracy of over 99%. Hasbullah et al. [20] designed a hybrid deep-learning model for MI detection from ECG signals. A 1D CNN was used to extract meaningful features from 1D ECG signals. The BiLSTM model was preferred for classification with these features. This model was evaluated on the PTB XL dataset, and a classification accuracy of 91% was achieved. Ayano et al. [21] utilized three deep-learning models to detect heart diseases such as MI, atrial fibrillation (AF), and CVD from ECG signals. First, features were extracted from the ECG signal with 1D-CNN. Then, these extracted features were transferred to two BiLSTM models. The tensors from the output of these models were imported into the 2D CNN model, and the classification was performed. This approach was evaluated on three different datasets. A classification accuracy of over 90% was achieved in all datasets. Latifoglu et al. [22] utilized a 12-channel ECG signal to detect heart attack. The ECG signal was used separately with the pre-trained AlexNet, ResNet, and VGG16 net models using a transfer learning strategy. In the next stage, the final classification results were obtained by applying the majority voting technique to the prediction results obtained from each model. Emin and Yüksel [23] used the characteristics of the ECG signal and patient demographics for automatic Coronary Artery Disease (CAD) detection. Stacked-Autoencoder (SA) was used to extract more meaningful features from these features. The SA algorithm extracted features at two levels. These extracted features were evaluated in a DNN model with two hidden layers. The softmax function was preferred in the classification process of the DNN model. This approach was evaluated on six different datasets available for use. This approach gave better results than classical machine learning methods.

Another part of the related studies in the literature was formed by using demographic data together with the analysed statistical values of ECG signals. These data were evaluated on classifiers used in machine learning. For CAD prediction, Omkari et al. [9] used ECG data and features such as pulse pressure, body mass index, mean arterial pressure, and demographic data. These data were obtained from two different datasets from the Kaggle and UCI databases. A two-tier voting strategy was adopted to improve the model’s predictive performance. Statistical methods in the first layer included ANOVA f-test, Chi-squared test, and Mutual Information. In the second layer, the prediction results of Multi-Layer Perceptron, Decision Tree (DT), SVM, and Random Forest (RF) algorithms were adjusted by the GridSearchCV algorithm. The classification accuracy was 99.03% for the UCI-based dataset and 88.9% for the Kaggle-based dataset. Rahman et al. [24] studied the UC Irvine Cleveland dataset, which uses analysis data of ECG signals and patient demographic data for CVD prediction. The dataset containing a total of 14 features was tested with the self-attention-based transformer model. This approach achieved a prediction accuracy of 96.51%. Yeh et al. [25] tried to detect the presence of CAD disease by using statistical values such as analysis values of ECG signals, demographic data such as age and gender, and the presence of chronic diseases such as diabetes. For this, the XGBoost classifier was utilised. In this study, a sensitivity of 84% was achieved.

Other existing studies in the literature are based on CAG images. Deep learning-based models have used these images to detect CAD and coronary artery occlusions. Jiménez-Partinen et al. [26] utilized CAG images to create an automatic CAD model. Different pre-trained CNN models such as DenseNet, MobileNet, NasNet, and ResNet were used for classification. The F-score and AUC values were 92.7% and 98.1%, respectively. Lee et al. [27] used a modified Inception V3 model to detect CAD from CAG images. In total, 7945 CAG images were used to evaluate the model’s performance. CAD was detected with 92.3% accuracy. Wang et al. [28] utilized a deep learning-based CNN model to detect coronary artery stenosis from CAG images. With this approach, 88.9% accuracy, 85.4% sensitivity, and 87.1% F-score performance values were obtained. Li et al. [29] used a hybrid depth learning model for CAD detection from CAG images. Deep neural network and recurrent neural networks were used for the hybrid model. The prediction results obtained from this model are enhanced with a KNN classifier. With this approach, an 82.8% accuracy, 88.57% sensitivity, and 87.82% F-score were obtained. Peng et al. [30] used CAG images in a transformer-based model. Thus, they achieved 81.5% sensitivity in detecting occlusions in the coronary artery.

### Gaps in the Literature, Motivation, and Contribution

When the literature is analyzed in detail, the studies on cardiovascular diseases are discussed in different sections. If the ECG signal is used in its raw form in a study, it is directed to problems such as detecting or classifying specific and general diseases such as AF, MI, and CVD. The area of interest of the proposed study is only the presence or absence of MI. If, in addition to analyzing values such as the RR peak value of the ECG signal and segment durations, information such as the patient’s age, gender, and the presence of chronic diseases are used, studies in this field have focused on the possibility that a patient may have occluded coronary arteries or cardiovascular disease. In another type of study, CAG images were used to determine which occluded coronary arteries were occluded.

As seen in the literature, no study classifies occluded coronary arteries using only ECG signals. Image data obtained after the CAG procedure were used to detect occluded coronary arteries. However, the CAG procedure is risky. The most important risks of the CAG procedure are internal bleeding, infection, stroke, heart attack, kidney damage, and radiation exposure. These risks are increased by advanced age and chronic diseases such as diabetes, hypertension, kidney disease, and blood clotting disorders [31,32,33]. For NSTEMI patients with small ST segment changes in ECG data, cardiologists cannot determine the necessity of the CAG group from ECG signals alone. In the existing literature, there is no study based on artificial intelligence or any other software application that detects the occluded coronary arteries of NSTEMI patients from ECG data. In addition, there is no dataset in the literature that would lead to a study in this field. In the general clinical procedure, the CAG procedure is applied to the vast majority of patients without differentiating between STEMI and NSTEMI patients and without considering the CAG risk status. This increases the workforce in the clinic and therefore increases the need for specialised personnel. At this point, there is a need for software applications that automatically find which main coronary arteries of patients are occluded from ECG data without the need for CAG for the NSTEMI patient group. This need was the motivation for this study. Firstly, a dataset that can be used in this field has been organized. A newly designed deep learning-based approach was used to classify the occluded main coronary artery on this dataset.

The main contributions of the proposed work are highlighted as follows:

A unique dataset containing 12-channel ECG signals of NSTEMI patients and their real label values has been created. The real label values, including occluded main coronary arteries, were generated by expert cardiologists. Thus, this unique dataset will be a study subject for other future researchers.

A new deep-learning modeling strategy has been developed. This model, named ConMixer, makes it possible to train each channel data of the 12-channel ECG signal simultaneously and to extract features with high discriminative power from these data.

In addition, an ensemble classification algorithm was developed that improves the performance of the SVM classifier according to feature importance weights.

## 2. Datasets

**Figure 1:**
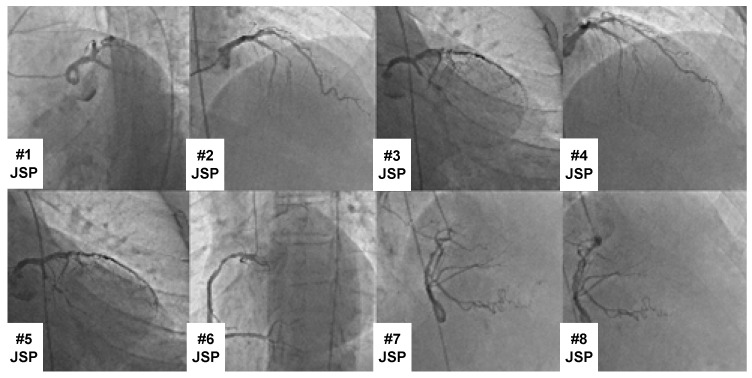
A CAG image with the label CX + LAD occluded following CAG processing.

Cardiologists at Fırat University gathered the dataset. Data were gathered using 12-channel digital ECG equipment. XML format was used to store the data, and 500 Hz and 1 KHz sample frequencies were used. The dataset’s primary coronary artery occlusions were designated as CX, RCA, and LAD. The designations CX + LAD, RCA + LAD, RCA + CX, and RCA + CX + LAD were used for several major coronary artery occlusions. Cardiologists used CAG for every ECG acquisition to ensure proper labeling. In addition, two different cardiologists performed the labeling separately to ensure labeling reliability. Different labeling errors were then resolved by agreement between the cardiologists. Following the CAG technique, a CAG image with the label CX + LAD obscured is displayed in Figure 1. This allowed for the recording and labeling of 1321 12-channel digital ECG signals. All of these ECG data were obtained from patients aged between 40 and 85 years. Of these patients, 592 were female and 729 were male.

**Figure 2:**
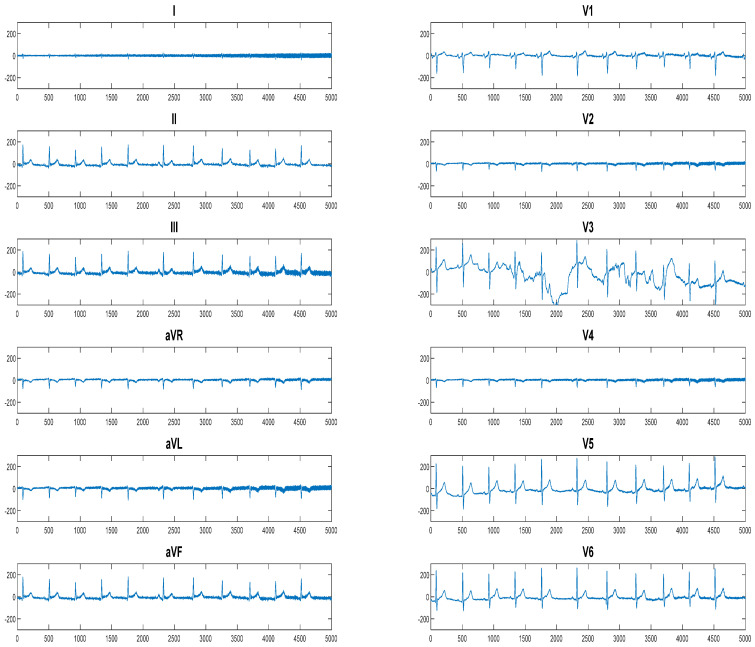
An intricate 12−channel ECG data illustration of the major coronary artery occluded by CX.

**Figure 3:**
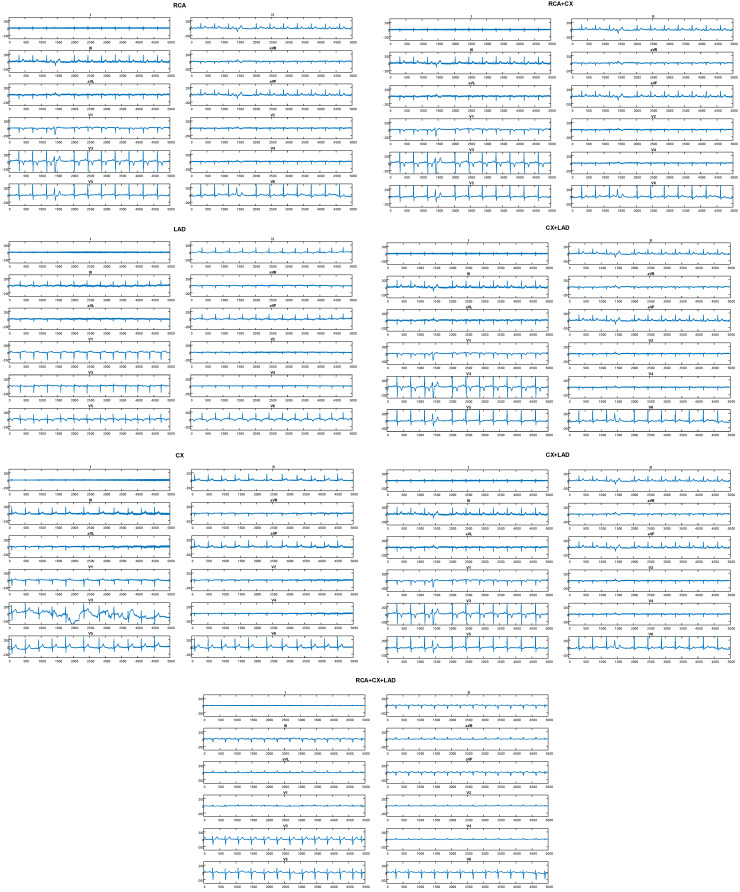
An illustration of each class’s sample digital ECG signal.

ECG signals from the CX class (180), RCA class (185), LAD class (252), CX + LAD class (183), RCA + LAD class (173), RCA + CX class (156), and RCA + CX + LAD class (192) are all included in the dataset. Figure 2 displays a comprehensive 12-channel ECG signal representation for the major coronary artery that is occluded by CX. Figure 3 displays an example of a digital ECG signal sample from each class.

## 3. Proposed Methodology

**Figure 4:**
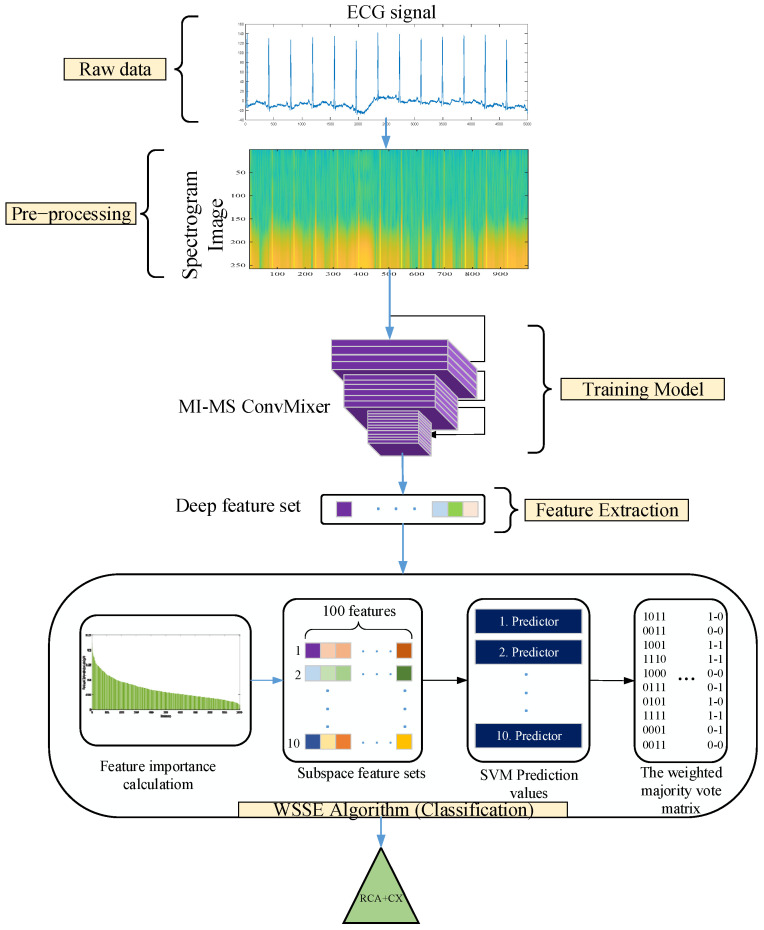
The framework of the proposed approach.

The proposed approach mainly consists of 4 basic steps. A representative illustration of the proposed approach is given in Figure 4. In the first stage, spectrogram transformations were performed to create time and frequency representations of ECG signals. Both 2D representations are achieved with spectrogram transformations, and the differences that may occur in the frequency axis are revealed [34]. In addition, the training infrastructure was started with 2D models, which are the most preferred and performant in deep learning models. In addition, the Viridis Colormap technique was also preferred to emphasize the pattern differences in spectrogram images [35].

**Figure 5:**
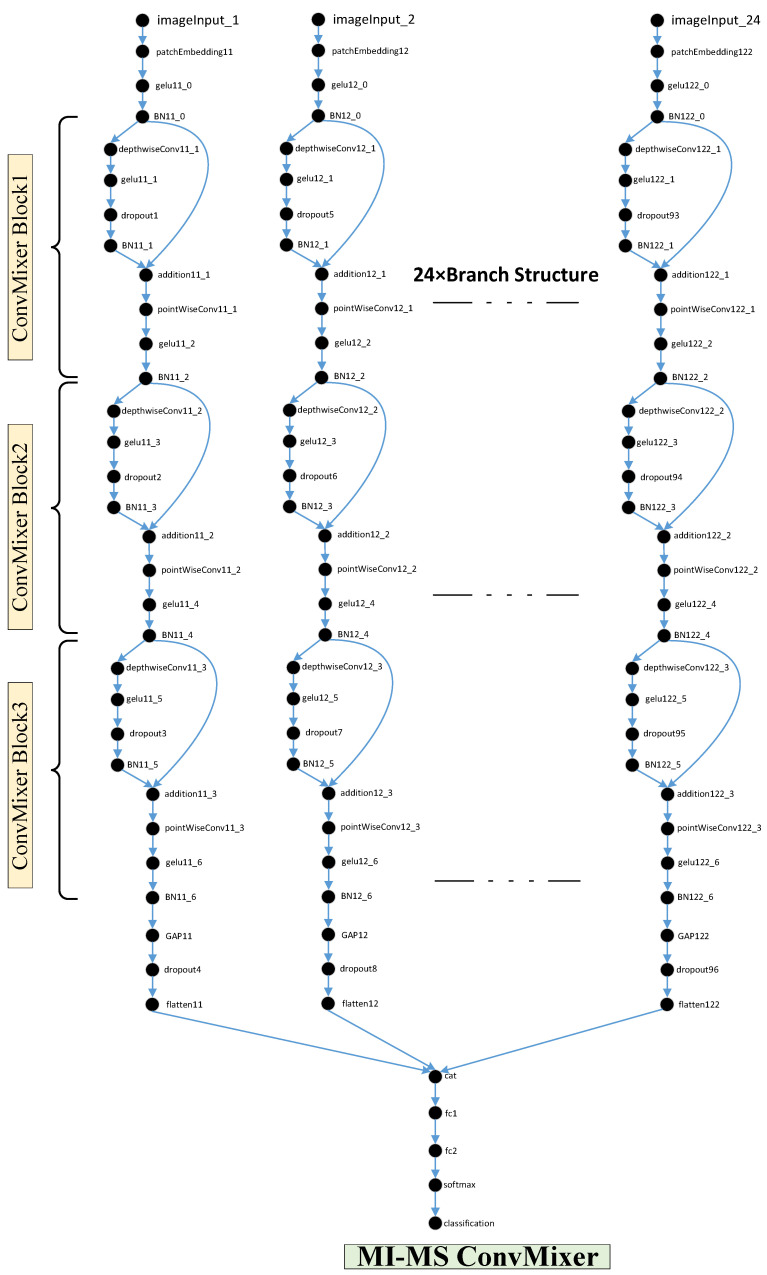
The layer structure of the MI-MS ConvMixer.

In the second stage, training was performed with a special Multi-Input and Multi-Scale (MI-MS) ConvMixer model. A representation of this model with its layer structure is shown in Figure 5. One of the key advantages of the model is that it has a multi-input (12 inputs) structure that allows simultaneous training of 12 spectrogram images obtained from the 12-channel ECG signal of each patient. The proposed MI-MS Convmixer model offers significant performance improvement contributions. Thanks to the multi-scale feature in the MI-MS ConvMixer model, the number of model inputs was increased to 24 by giving different scale scales in the 12-input structure, and more focus was provided on the details of the spectrogram images. Also, the MI-MS Convmixer model offers the possibility of combining convolution and linear mixing. Thus, both local features and the relationship between different channels can be learned. In addition, the MI-MS Convmixer model has fewer parameters to be learned than pure transformer models. This makes the MI-MS Convmixer model efficient even on small datasets.

**Figure 6:**
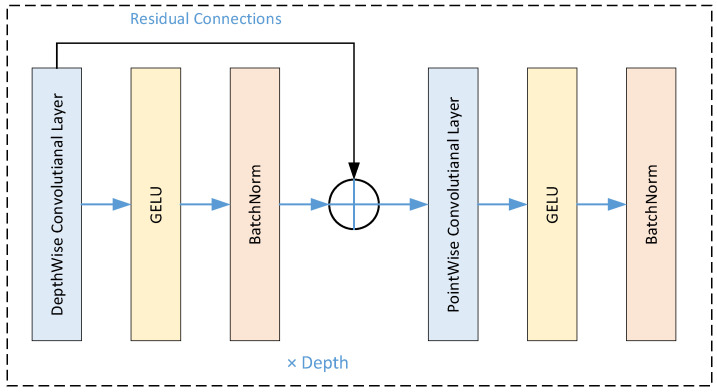
The block diagram of a ConvMixer internal structure.

The internal structure of a ConvMixer model is given in Figure 6. The MI-MS ConvMixer model is designed for the classification of 12-lead ECGs with spectrogram images derived from the ECG signals. The spectrograms can be represented as input tensors of size $X\in R^{64\;\times \;64\;\times \;3}$ where 64 × 64 is the spatial resolution and 3 corresponds to the number of color channels [36,37]. A non-overlapping sliding window is used for dividing the input tensor into the patches:

(1) $$X_{patch}=Conv2D\left(X,W_{patch},b_{patch},stride=P\right)$$

where *P* shows the patch size, $W_{patch}\in R^{P\;\times \;P\;\times \;3\;\times \;H}$ is the convolutional kernel, $b_{patch}$ is the bias, and *H* is the hidden dimension [38]. As this is a multi-scale approach, the patch size is selected as both 16 and 8, and the *H* is chosen as 750. Patching operation reduces the spatial resolution and projects the patches into a high-dimensional feature space. The resulting feature map is processed as

(2) $$X_{embed}=BatchNorm\left(GeLU\left(X_{patch}\right)\right)$$

A depthwise convolutional layer combined with residual connections is employed to embed each patch. For a depth *i*, the transformation is

(3) $$X_{i}=BatchNorm\left(GeLU\left(DepthwiseConv2D\left(X_{i-1},W_{depthwise,i}\right)\right)\right)+X_{i-1}$$

where $W_{depthwise,i}\in R^{K\;\times \;K\;\times \;H}$ is the kernel for depthwise convolution, and *K* is the kernel size. The *K* value is selected as 3 [39]. Mixing the features across all the channels is carried out with a pointwise convolution operation:

(4) $$X_{proj,\;i}=BatchNorm\left(GeLU\left(Conv2D\left(X_{i},W_{pointwise,i}\right)\right)\right)$$

where $W_{pointwise,i}\in R^{1\;\times \;1\;\times \;H\;\times \;H}$. A global average pooling layer is employed after the pointwise convolution layer to reduce the number of features [40]. This operation is shown in the following equation:

(5) $$X_{GAP}=GlobalAveragePooling2D\left(X_{proj,\;D}\right)$$

where *D* is the total depth of the ConvMixer layer [41,42,43]. The final concatenated features from multiple scales are shown:

(6) $$X_{concat}=Concat\left(X_{GAP,1},\ldots ,X_{GAP,S}\right)$$

where *S* shows the number of scales. The concatenated features are classified over a fully connected layer where the Softmax activation function is used:

(7) $$\hat{y}=Softmax\left(W_{fc}X_{concatGAP}+b_{fc}\right)$$

where $W_{fc}$ and $b_{fc}$ are the weight and bias of the fully connected layer, respectively.

### Superiority of the Proposed Approach to Technical Analysis

To provide a comprehensive evaluation, the performance of the MI-MS ConvMixer model was compared with other state-of-the-art methods such as standard CNN and BiLSTM architectures. While CNN models excel in extracting spatial features and have demonstrated high specificity in identifying single coronary artery occlusions, their performance tends to decline in scenarios involving multiple occlusions due to their limited ability to model long-range dependencies across features. In contrast, BiLSTM models are highly effective in capturing sequential patterns and temporal dependencies, particularly in datasets with ordered inputs or time-series data. However, BiLSTMs are computationally intensive, require extensive training times, and may underperform in datasets where spatial feature extraction is critical. The MI-MS ConvMixer model combines the strengths of both approaches by employing a multi-input, multi-scale architecture that integrates spatial and contextual features more effectively. In addition, the proposed model gives better performance on medium-sized datasets with fewer parameters than models such as ResNet and VİT.

The WSSE algorithm used in the proposed approach focuses on improving the classification performance of algorithms such as SVM and KNN used in machine learning. The SVM algorithm was preferred in the study because it is the most widely used in the literature and provides good performance in classification problems. The WSSE algorithm allows it to give more weight to the distinctive ones rather than all of the extracted features, and therefore to be more effective in the estimation results. The ReliefF algorithm, which is both effective and does not require much computational cost, was used in feature weighting.

**Figure 7:**
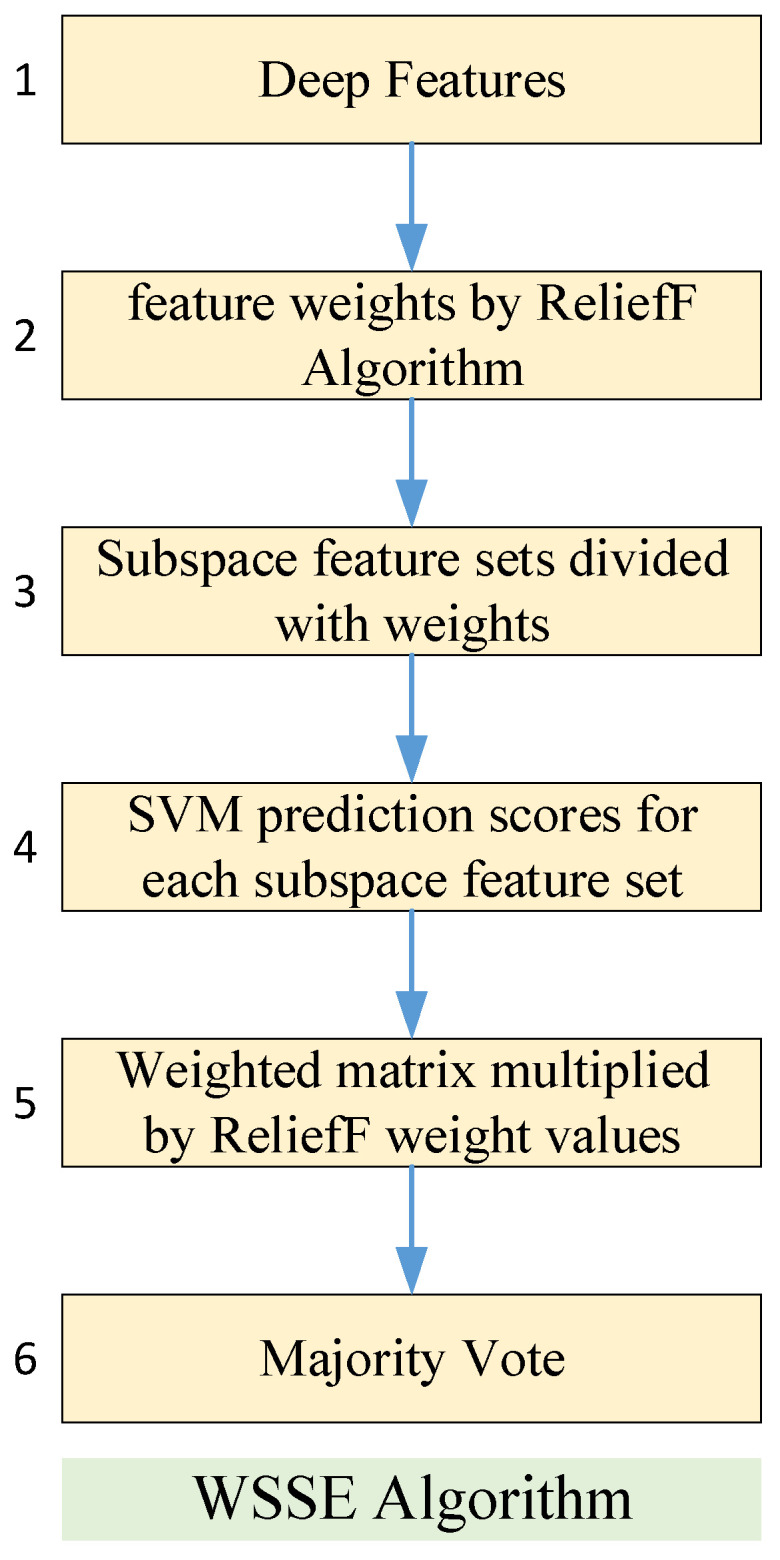
The block diagram of the WSSE algorithm.

In the third stage, features (1000 features) are extracted from the layer fc1 of the MI-MS ConvMixer model of the proposed approach to further improve the classification performance. In the fourth stage, the weighted subspace SVM ensemble algorithm (WSSE) weighted by feature importance is developed to achieve high classification performance. Algorithm 1 presents the pseudocode of the VSSE algorithm. Figure 7 summarizes the pseudocode in Algorithm 1. In the first step of the WSSE algorithm, the importance weights of each feature in the feature set are calculated by the ReliefF algorithm [44]. The ReliefF algorithm performs particularly well on complex and multidimensional data. In addition, while measuring the relationship of a feature with the target variable, it also takes into account the correlation of that feature with other features [45,46]. The features are ranked according to their importance weights in the next step. Then, the feature set ranked by a specified value is divided into subspaces. For each subspace, classification prediction results are assigned to a matrix by the SVM algorithm. A weighted majority voting process is applied to this matrix. For example, since the prediction results in the first subspace matrix have the highest feature importance weight, they will have the highest weight in the majority voting. This weighting is conducted in the majority voting matrix. The prediction results with the highest weights have the most repeated values in the majority voting matrix. Thus, the WWSE algorithm improves the classification capability compared with a known SVM algorithm.
**Algorithm 1.** Pseudocode of the WSSE algorithm.**Algorithm WVWSE (Weighted Voting Subspace Ensemble)** **Input:** Feature set F, Label set Y, Subspace division parameter k **Output:** Final classification prediction P_final_Compute feature importance weights using ReliefF:For each feature f in F:○Compute weight W[f] using ReliefF algorithm.**Sort features based on importance weights:**○Sort F in descending order of W.**Divide sorted feature set into k subspaces:**○Divide F into k subspaces (S_1_, S_2_, …, S_k_) where each subspace contains a subset of features.**Classify each subspace using SVM:**○Initialize matrix M to store classification predictions.○For each subspace S_i_ in (S_1_, S_2_, …, S_k_):▪Train an SVM model using features in S_i_ and labels in Y.▪Predict class labels for S_i_ using the trained SVM model.▪Store prediction results in matrix M at row i.**Apply weighted majority voting:**○Initialize a weighted voting matrix V.○For each prediction result in M:▪Assign weights to predictions based on the importance of features in the corresponding subspace.▪Update V with weighted predictions.**Determine final predictions:**○For each sample in V:▪Choose the most frequent class with the highest weight.▪Assign this class as the final prediction.**Return P_final_ as the final classification predictions.**

## 4. Experimental Studies

**Figure 8:**
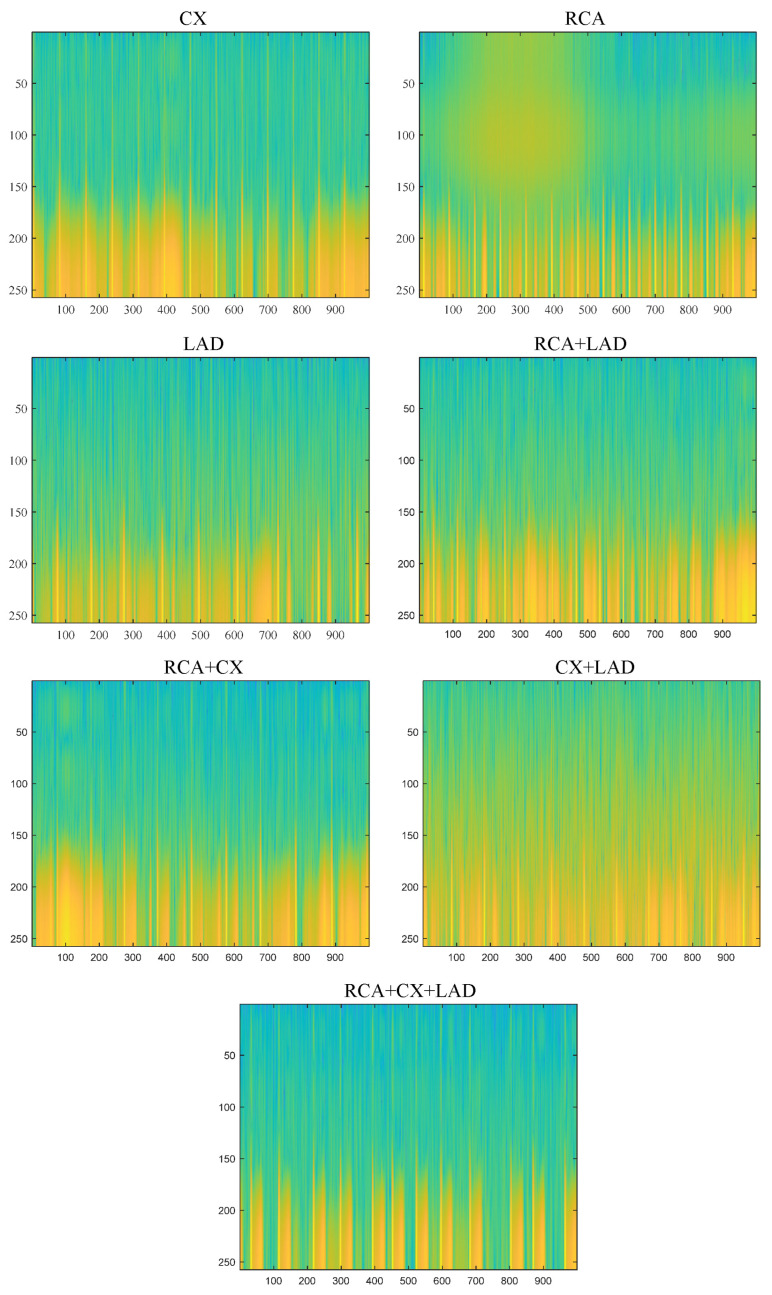
Spectrogram images obtained from ECG signals for each class.

**Figure 9:**
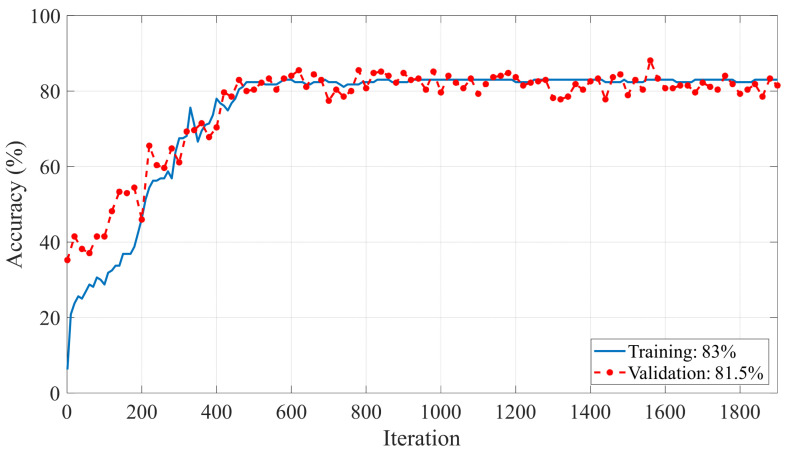
Accuracy graphs of the training-validation process for the MI-MS ConvMixer model.

**Figure 10:**
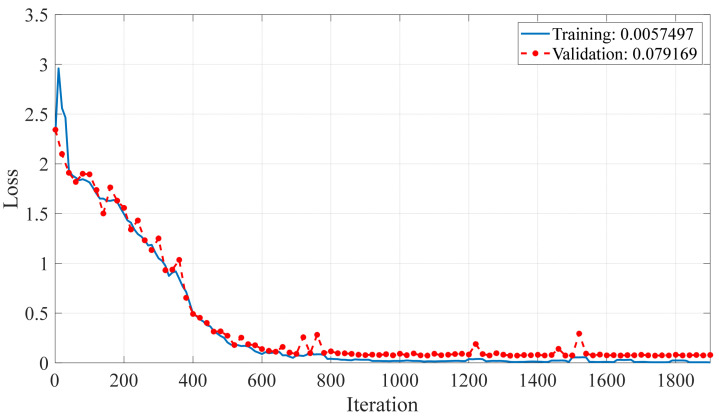
Loss graphs of the training-validation process for the MI-MS ConvMixer model.

In this study, the Matlab program installed on the Windows 11 operating system installed on computer hardware with an i9 intel processor (13th generation), 64 GB of DDR5 memory, and 24 GB of GDDR6 capacity (NVIDIA RTX4090 ASUS) was used to perform experimental studies. The equipment was sourced from Intel Corporation in Santa Clara, United States, for the i9 processor (13th generation); NVIDIA Corporation in Santa Clara, United States, and ASUS in Taipei, Taiwan, for the NVIDIA RTX 4090 graphics card; and a manufacturer such as Corsair for the 64 GB of DDR5 memory. In order to obtain spectrogram images from the raw data, all of the raw ECG data in XML format were first read and visualized. Before spectrogram conversion, each ECG signal and its ST segments were analyzed by cardiologists. If the signals contained network noise or noise from the electrodes, these data were excluded from the evaluation. Then, windowing type and size, Fast Fourier transform length, and sampling frequency values were adjusted for the spectrogram conversion of the ECG signal in each class. Hamming windowing rounds the edges of the signal and thus allows better discrimination in the frequency domain [47,48]. The window size was reduced to a certain value until a good resolution was achieved in the spectrogram images. To obtain the spectrogram data from the raw ECG data, the Hamming size of the windowing function in the spectrogram transformation was chosen as 10. The sampling frequency was set to 500 Hz and the FFT length to 512. Figure 8 shows the images obtained by spectrogram transformations for each class in the generated dataset. There are pattern and color differences for each class. This increases the discrimination ability on the classification problem. For the training of the MI-MS ConvMixer model, the data were organized according to the 10-fold cross-validation technique. Each data point is tested with the K-fold cross-validation technique. As a result, at the end of the training of the model, the generality and therefore the reliability of the model for the classification problem increase. Thus, the sensitivity to the overfitting problem is minimized. The value of K was chosen because the most commonly used value in the literature is 10 [49,50]. In the training options of this model, the number of epochs was selected as 100, the mini-batch value as 128, the initial learning rate as 0.001, and the validation frequency as 30. The updating of the weights was optimized with the SGDM algorithm. The initial learning rate of 0.001 was chosen because it is a value frequently used in deep learning models [51]. When the initial chosen learning rate is too large, there is a possibility of moving away from the global minimum points in the optimisation process. When it is chosen too small, both the training process is prolonged and the tendency of the model to overfitting increases. The mini batch size was chosen as 128 based on twice the default value. This prevents fluctuations in the accuracy change graph during the training process. The number of epochs was also set to a sufficient value to stabilise the accuracy and loss values, as shown in Figure 9 and Figure 10.

Figure 9 and Figure 10 show the accuracy loss variation plots for both the training and validation of the MI-MS ConvMixer model over 1900 iterations. At the end of 1900 iterations, the training and validation accuracies were 83% and 81.5%, respectively. At the end of 1900 iterations, the training and validation loss values were 0.0057497 and 0.079161, respectively.

**Figure 11:**
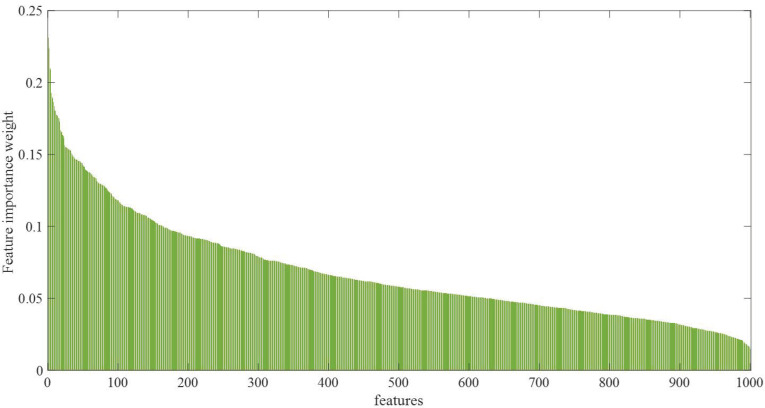
Weight value graphs calculated with the ReliefF algorithm for feature importance degrees.

After training the MI-MS ConvMixer model, weights were recorded. Using these recorded weight values, 1000 features were extracted from the fully connected layer of the model named fc4, and a feature set was created. The ReliefF algorithm was used to identify the features with strong discriminative features. In the ReliefF algorithm, the number of nearest neighbors is set to 10, the prior probability selection option for each class is empirical, and the sigma value is set to 20. Weights of 1000 features were calculated with the ReliefF algorithm. Figure 11 shows the weights calculated from the highest weight value to the lowest weight value. The highest weight value is 0.0907, the lowest weight value is 0.03, and the average weight value is 0.0628.

## 5. Discussion

In this section, ablation studies were performed to demonstrate the effectiveness of the proposed approach. Table 1 compares the MI-MS ConvMixer model and popular pre-trained deep learning models. The comparison shows the models’ layer size, input data size, hardware memory footprint, and classification performance. Table 1 provides information on 17 different models. The model with the highest number of layers is densenet201 (201 layers), and the model with the lowest number of layers is AlexNet (eight layers). The MI-MS ConvMixer Model ranks third in the number of layers with 148 layers. The model with the highest model memory is vgg19 (548 MB), and the model with the lowest is MI-MS ConMixer (158 KB). The MI-MS ConvMixer Model has the lowest memory size, about 30 times less than the Squeezenet pre-trained model. When looking at the input size of the pre-trained models, it is seen that 224 × 224 is generally preferred. However, Xception and Inception models use larger input sizes, such as 299 × 299. All models were evaluated on the dataset we created in light of this information. In this study, 10-fold cross-validation was preferred as the evaluation criterion. The results show that the MI-MS ConvMixer model achieved the best classification performance (81%), while the Xception model performed the worst (59%). Moreover, the ConvMixer model achieved this performance with a very low memory size and lower resolution. This shows the efficiency of the designed ConvMixer model.

Table 2 shows the performance of the features extracted from the first fc layer of the MI-MS ConvMixer model on popular classifiers used in machine learning. Naive Bayes, Logistic Regression, Decision Tree, KNN, Random Forest, and SVM algorithms were used for classification. This experimental phase aimed to select the best classification algorithm for the WSSE algorithm. As can be seen from Table 2, the SVM algorithm gave the best classification performance, while the Naive Bayes algorithm gave the worst classification performance.

**Figure 12:**
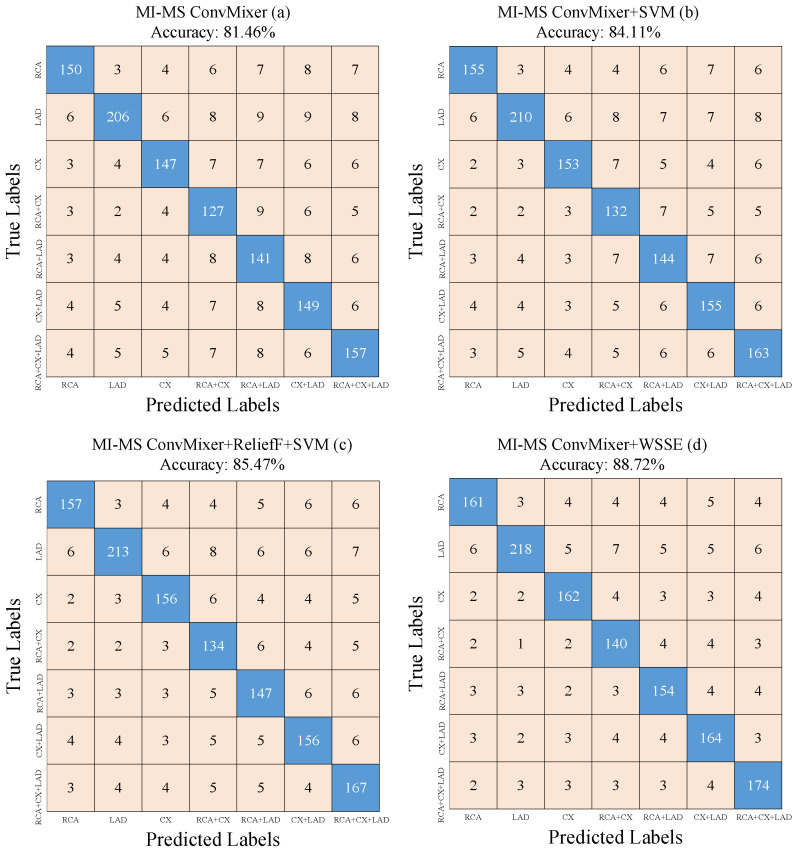
Ablation studies for the effectiveness of the WSSE: (**a**) MI-MS CovMixer confusion matrix, (**b**) MI-MS CovMixer+SVM confusion matrix, (**c**) MI-MS CovMixer+ReliefF+SVM confusion matrix, (**d**) MI-MS CovMixer+WSSE confusion matrix.

Figure 12 shows the results of the confusion matrices from the ablation studies to demonstrate the effectiveness of the WSSE algorithm. Figure 12a shows the confusion matrix results obtained after the end-to-end training of the MI-MS ConvMixer model. Figure 12b shows the confusion matrix results after the classification process obtained by transferring the features extracted from the first fc layer of the MI-MS ConvMixer model to the SVM algorithm. The confusion matrix in Figure 12c is given to compare the WSSE algorithm and the ReliefF algorithm used in the WSSE algorithm. Figure 12d shows the confusion matrix results of the proposed approach. Table 3 is obtained by using the confusion matrix values in Figure 12. Table 3 shows the accuracy, sensitivity, specificity, precision, and F-score metrics.

The performance metrics values obtained from the MI-MS ConvMixer model ranged between 0.81 and 0.82 for each class. It is seen that the success of the model in capturing the true positive (TP) samples is around 80%. Specificity values were calculated between 0.95 and 0.98 in seven classes. Thus, the MI-MS ConvMixer model provided a balanced and high-level performance in predicting true negative (TN) samples for each class. Precision values ranged between 0.9 and 0.75. More unstable values were observed compared with previous metrics. This indicates that false positive (FP) sensitivity between classes is low. F-score values were analyzed to examine the balance between FP and TP. The F-score value was between 0.86 and 0.78. Table 3a shows that the F-score value was higher in the classes with single coronary artery occlusion. In the case of Table 3b, the sensitivity value for each class was improved between 0.2 and 0.3 with the SVM classifier, and the balance between the classes was maintained. Regarding the specificity value, the values remained the same for some classes, while there was an increase of 0.1 in other classes. When the precision value was analyzed, the performance was improved by 0.4 and 0.5 (RCA + CX and RCA + LAD) in the classes with the lowest value. In other classes, performance was improved by 0.1 and 0.3. In the F-score metric, the performance was improved between 0.1 and 0.4, and the balance between the classes increased.

To further emphasize the effectiveness of the WSSE algorithm in Table 3c, the ReliefF algorithm used in the WSSE algorithm was used only for the feature selection task. Then, the SVM algorithm was used for classification. With this technique, the sensitivity metric improved between 0.1 and 0.2. The specificity metric improved by 0.1 in coronary arteries with bilateral occlusions. In the precision metric, performance was improved in all coronary arteries with multiple occlusions. In the F-score metric, performance was improved between 0.1 and 0.2 in all classes. In Table 3d, compared with Table 3c, the sensitivity metric improved between 0.1 and 0.5 in all classes. The precision metric improved by 0.1 for the LAD class. A performance value of 0.98 was achieved for all other multiple main coronary occlusions. Performance improved between 0.2 and 0.5 for all classes in the precision metric. The F-score metric improved between 0.1 and 0.4 for all classes.

**Figure 13:**
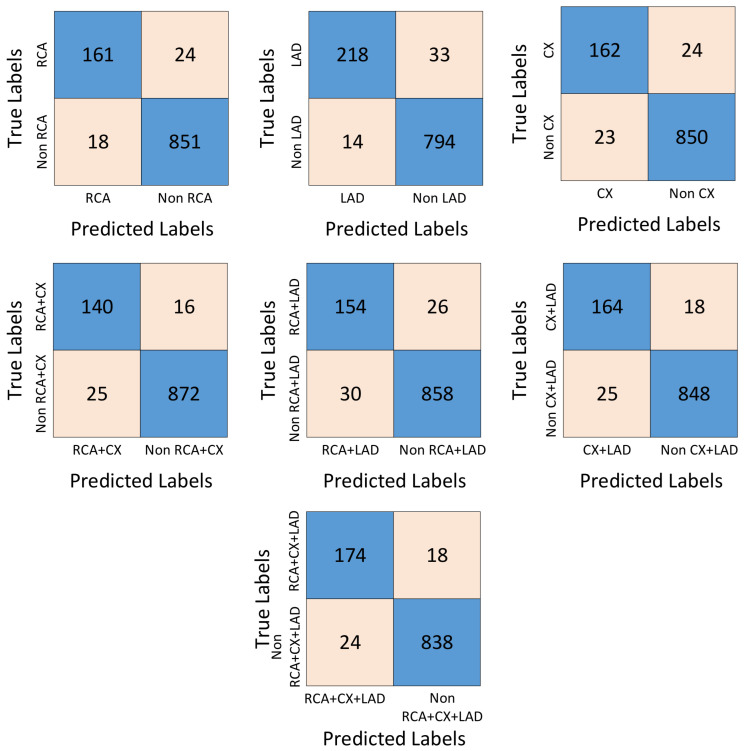
Confusion matrix results of the proposed approach by classes.

Figure 13 presents the confusion matrices for the MI-MS ConvMixer + WSSE model, showcasing its classification performance across all individual and combined coronary artery classes. For the RCA class, the model achieved 161 true positives and 851 true negatives, with relatively low false positives (24) and false negatives (18), indicating reliable detection. Similarly, for the LAD class, the model produced 218 true positives and 794 true negatives, alongside 33 false positives and 14 false negatives, demonstrating strong sensitivity and precision. The CX class displayed comparable performance, with 162 true positives, 850 true negatives, 24 false positives, and 23 false negatives, reflecting robust classification accuracy.

In terms of combined conditions, the RCA + CX class recorded 140 true positives and 872 true negatives, with just 16 false positives and 25 false negatives, maintaining a high balance between sensitivity and specificity. The RCA + LAD class achieved 154 true positives and 858 true negatives, alongside 26 false positives and 30 false negatives, showcasing consistent performance. For the CX + LAD class, 164 true positives and 848 true negatives were observed, with minimal false positives (18) and false negatives (25). Finally, the RCA + CX + LAD class achieved the highest overall true positive rate (174) and true negatives (838), with relatively few false positives (18) and false negatives (24), indicating the model’s strong capability to classify more complex cases involving multiple coronary artery conditions.

The WSSE algorithm demonstrated the most significant reduction in misclassification rates. Sensitivity improvements of up to 0.05 in complex classes such as RCA + CX and RCA + LAD reflect fewer false negatives, while precision improvements (up to 0.05) across these classes suggest a significant reduction in false positives. This highlights the effectiveness of WSSE in addressing the inherent feature overlap between these complex classes and improving overall classification accuracy.

Classes containing multiple coronary artery occlusions (e.g., RCA + CX, RCA + LAD, CX + LAD) are more prone to misclassification due to the inherent overlap in feature patterns between these classes and single occlusion classes. For example, occlusions in the RCA and CX arteries may exhibit similar feature distributions, leading to false positives in the RCA + CX class. Similarly, the presence of ambiguous or less distinguishable patterns in multiple occlusion scenarios increases the likelihood of false negatives. Model refinements through SVM, ReliefF, and WSSE show that better feature prioritization and improved classification methods can address these challenges, but further improvements are required due to the complexity of multiple congestion patterns.

The performance metrics values obtained from the MI-MS ConvMixer model ranged between 0.81 and 0.82 for each class, with 95% confidence intervals (CIs) calculated for each metric to ensure statistical validity. For instance, sensitivity values for individual classes were found to range from 0.78 to 0.85 (95% CI), indicating a robust model performance in identifying true positive (TP) samples. Specificity values were calculated between 0.95 and 0.98 across seven classes, with 95% CI values confirming the consistency of the model in identifying true negative (TN) samples. Precision values ranged between 0.75 and 0.90, and although more unstable than other metrics, the inclusion of confidence intervals, such as 0.70–0.80 for certain classes, highlights the model’s potential variability in false positive (FP) sensitivity. F-score values ranged between 0.78 and 0.86, with confidence intervals further demonstrating that performance was higher in classes with single coronary artery occlusions (Table 3a). Similarly, the use of the SVM classifier (Table 3b) resulted in improvements in sensitivity (by 0.2–0.3), specificity (by up to 0.1), and precision (by 0.4–0.5 in certain classes). These improvements are validated with CIs indicating statistical reliability. Further analysis of Table 3c,d using the WSSE algorithm confirmed significant gains in sensitivity, specificity, precision, and F-score metrics, all of which are reported with 95% confidence intervals to validate the robustness of the results.

When the performance values of the proposed approach are analyzed (Table 3d), higher performance is obtained in classes with single coronary artery occlusions than in classes with multiple coronary artery occlusions. This study aimed to detect occluded coronary arteries in ECG data and determine the CAG requirement in NSTEMI patients. CAG can recommend drug therapy to cardiologists in patients with occlusion of single coronary arteries and in patients in whom the CAG procedure is risky. From this point of view, high-performance prediction in single main coronary arteries strengthens the hand of cardiologists in decision support. On the other hand, the lowest performance in multiple main coronary arteries was calculated as 0.87. This value is also a good value.

The MI-MS ConvMixer model demonstrates not only high classification performance but also significant computational efficiency, making it highly suitable for real-world applications. With 81 layers, the model has a compact size of only 158 KB, which is considerably smaller compared with many state-of-the-art deep learning models such as ResNet or BiLSTM architectures, which often exceed several megabytes in size. This compactness reduces memory requirements, enabling deployment on resource-constrained devices, such as portable medical systems or edge devices in clinical settings.

In terms of runtime efficiency, the MI-MS ConvMixer was designed to balance depth and computational complexity, allowing for faster inference times. When tested on a standard GPU setup (e.g., Nvidia Tesla V100(The Nvidia Tesla V100 was sourced from NVIDIA Corporation, located in Santa Clara, CA, USA))**,** the average inference time per sample was approximately 2 ms, making it capable of real-time analysis. This speed is particularly advantageous for clinical scenarios requiring quick decision-making, such as automated coronary artery occlusion detection during patient monitoring.

The combination of a small memory footprint, fast inference time, and reduced training requirements underscores the model’s potential for real-world deployment in healthcare systems, particularly where computational resources are limited or rapid response times are critical.

The MI-MS ConvMixer model, while effective on internal datasets, faces limitations on external datasets due to sensitivity to domain shifts, class imbalances, and overfitting to training data. These challenges may result in reduced generalization, higher false positive/negative rates, and limited robustness to diverse imaging conditions. Additionally, the model’s compact size (158 KB), while computationally efficient, may restrict scalability to larger datasets, and its “black-box” nature limits interpretability in clinical applications. Addressing these limitations requires incorporating transfer learning, domain adaptation, dataset diversification, and explainability techniques to improve generalization and real-world applicability.

The MI-MS ConvMixer model, trained on a specific dataset, may face challenges when generalized to different demographics or ECG devices due to variations in signal characteristics, such as frequency, noise, and amplitude, as well as demographic factors like age, gender, and health conditions. To enhance generalization across these variables, the model could benefit from data augmentation techniques, such as adding synthetic variations of ECG signals to mimic different demographics and device outputs. Furthermore, fine-tuning the model with diverse datasets from various ECG devices and demographics, as well as employing domain adaptation strategies, could improve its robustness.

## 6. Conclusions

In this study, we focused on heart attack and coronary artery occlusion, which is a very important issue in human health. NSTEMI patients with a history of heart attacks formed the basis of the study. The main motivation for this study was to detect occluded main coronary arteries from ECG signals in NSTEMI patients without CAG processing using a novel deep learning-based method. First, a unique dataset containing 12-channel ECG signals of NSTEMI patients was created with the help of expert cardiologists. The occluded coronary artery or arteries of each ECG signal were labeled by these cardiologists using the CAG process. Next, deep learning-based software applications were developed for the automatic detection of occluded main coronary arteries in NSTEMI patients. The MI-MS ConvMixer model was designed and trained as a deep-learning model. The performance efficiency of this model is compared with popular lifetime-trained CNN models, and the superiority of the proposed model is proved by experimental studies. In the next stage, to further improve the classification performance, features are extracted in the first fc layers of the MI-MS ConvMixer model. These extracted features are classified with the newly developed WSSE algorithm. This algorithm improves the classification performance of the SVM classifier. In addition, the performance efficiency of the WSSE algorithm is demonstrated by ablation studies. The proposed approach correctly predicted the occluded main corneal artery or arteries of NSTEMI patients with an accuracy of 88.72%. Thus, an important study has been conducted for drug treatment methods without CAG procedures, especially for NSTEMI patients in whom CAG procedures are risky. Drug therapy will be preferred for elderly patients, patients with chronic diseases, and patients with occlusion of a single main coronary artery. In addition, this study will be a baseline model for other researchers in the future, and new models will be developed based on the dataset generated in this study. Thus, decision support systems with higher reliability will be used more and more in the field of cardiology.

This study demonstrates the potential of the MI-MS ConvMixer model in advancing ECG signal classification for diagnosing coronary artery diseases, offering a promising tool for real-time, automated analysis in clinical settings. The findings highlight the model’s ability to achieve high sensitivity, specificity, and precision, which could significantly improve diagnostic accuracy and reduce clinician workload. The translational potential of this work lies in its capacity to be integrated into clinical workflows, where it could aid in decision-making by providing quick and reliable diagnostics, particularly in emergency situations. Future research could focus on expanding the model’s application to diverse patient populations and ECG devices, ensuring its robustness across different settings. Additionally, this study paves the way for exploring the use of deep learning models in other diagnostic domains, encouraging further innovations in automated healthcare solutions. By reducing dependency on manual interpretation and increasing diagnostic speed, the model could have a profound impact on clinical practices, ultimately improving patient outcomes and healthcare efficiency.

## Figures and Tables

**Table 1:** Comparison of MI-MS ConvMixer Model with Popular Pre-trained Models.

Model	Layer Number	Model Memory	Input Size	Accuracy (%)
squeezenet	18	4.7 MB	227 × 227	76
googlenet	22	27 MB	224 × 224	68
inceptionv3	48	91 MB	299 × 299	64
densenet201	201	77 MB	224 × 224	63
mobilenetv2	53	14 MB	224 × 224	79
resnet18	18	45 MB	224 × 224	75
resnet50	50	98 MB	224 × 224	77
resnet101	101	171 MB	224 × 224	62
xception	71	88 MB	299 × 299	59
inceptionresnetv2	164	213 MB	299 × 299	66
shufflenet	50	5.5 MB	224 × 224	63
darknet19	19	80 MB	256 × 256	62
darknet53	53	159 MB	256 × 256	68
efficientnetb0	82	20 MB	224 × 224	74
alexnet	8	233 MB	227 × 227	73
vgg16	16	528 MB	224 × 224	79
vgg19	19	548 MB	224 × 224	77
MI-MS ConvMixer	148	158 KB	100 × 100	81

**Table 2:** Comparison of Classifier Algorithms.

Classifier	Accuracy (%)
KNN	83.20
Naïve Bayes	80.93
Logistic Regression	81.31
Decision Tree	81.08
SVM	84.11
Softmax	81.46
Random Forest	82.02

**Table 3:** Ablation studies for performance WSSE algorithm.

MI-MS ConvMixer (a)					MI-MS ConvMixer + SVM (b)				
Class	Sensitivity	Specificity	Precision	F-score	Class	Sensitivity	Specificity	Precision	F-score
RCA	0.81	0.98	0.87	0.84	RCA	0.84	0.98	0.89	0.86
LAD	0.82	0.97	0.90	0.86	LAD	0.83	0.98	0.91	0.87
CX	0.82	0.97	0.84	0.83	CX	0.85	0.98	0.87	0.86
RCA + CX	0.81	0.96	0.75	0.78	RCA + CX	0.85	0.96	0.79	0.81
RCA + LAD	0.81	0.95	0.75	0.78	RCA + LAD	0.83	0.96	0.80	0.81
CX + LAD	0.81	0.96	0.78	0.79	CX + LAD	0.85	0.96	0.81	0.83
RCA + CX + LAD	0.82	0.96	0.81	0.81	RCA + CX + LAD	0.85	0.96	0.82	0.83
**MI-MS ConvMixer + ReliefF + SVM (c)**					**MI-MS ConvMixer + WSSE (d)**				
Class	Sensitivity	Specificity	Precision	F-score	Class	Sensitivity	Specificity	Precision	F-score
RCA	0.85	0.98	0.89	0.87	RCA	0.87	0.98	0.90	0.88
LAD	0.85	0.98	0.92	0.88	LAD	0.87	0.99	0.94	0.90
CX	0.87	0.98	0.87	0.87	CX	0.90	0.98	0.90	0.90
RCA + CX	0.86	0.97	0.80	0.83	RCA + CX	0.90	0.98	0.85	0.87
RCA + LAD	0.84	0.97	0.83	0.84	RCA + LAD	0.89	0.98	0.86	0.87
CX + LAD	0.85	0.97	0.84	0.85	CX + LAD	0.90	0.98	0.87	0.88
RCA + CX + LAD	0.87	0.96	0.83	0.85	RCA + CX + LAD	0.91	0.98	0.88	0.89

## Data Availability

Data available on request due to restrictions.
